# The Minimum Dietary Diversity for Women (MDD-W) Score: Its Association With the Prevalence and Severity of Anemia in Pregnancy

**DOI:** 10.7759/cureus.66248

**Published:** 2024-08-05

**Authors:** Nishal Sharma, Jugal Kishore, Monika Gupta, Himal Singla, Rohini Dayma, Jai Bhagwan Sharma

**Affiliations:** 1 Community Medicine, Vardhman Mahavir Medical College and Safdarjung Hospital, New Delhi, IND; 2 Obstetrics and Gynaecology, Vardhman Mahavir Medical College and Safdarjung Hospital, New Delhi, IND; 3 Obstetrics and Gynaecology, All India Institute of Medical Sciences, New Delhi, New Delhi, IND

**Keywords:** prevelence, minimum dietary diversity for women (mdd-w) score, severity, aneamia, pregnancy

## Abstract

Background: Anemia during pregnancy is a very common medical disorder and is usually related to poor dietary nutrients.

Objective: The objective of this study was to study the Minimum Dietary Diversity for Women (MDD-W) score during pregnancy and its correlation with the prevalence and severity of anemia in a tertiary referral hospital.

Material and method: A total of 430 women in their second and third trimesters of pregnancy were included and details of sociodemographic, obstetric, and nutritional factors were asked from all participants. MDD-W score was calculated and was correlated with the prevalence and severity of anemia.

Results: The mean age, median parity, and mean body mass index were 26.2 years, 2, and 22.4 kg/m^2^,respectively. Of the patients, 30% were in lower socioeconomic classes. Anemia was seen in 250 (48.84%) women, being mild in 25.81%, moderate in 15-8%, and severe in 7.04%. A total of 49.3% of patients were vegetarian. The mean dietary calories and protein and iron intake were less in anemic patients. MDD-W score was 6.2±1.2 in the normal hemoglobin group, which was significantly higher than the anemia group (3.8±0.75). The mean MDD-W score was 4.4±0.9 in mild anemia, 3.5±0.7 in moderate anemia, and only 2.2±0.45 in severe anemia.

Conclusion: The MDD-W score was significantly less in anemic pregnant patients, being least in patients with severe anemia.

## Introduction

Anemia during pregnancy is the commonest medical disorder, especially in developing countries like India [1-3]. The prevalence of anemia in pregnancy is nearly 38.2% globally, 14% in developed countries, 51% in developing countries, and ranges from 65% to 95% in studies reported in India [4-7]. The National Family Health Survey-5 (NFHS-5) [8] observed a prevalence of anemia in pregnancy of 52.2% in 2019-2021, as compared to 50.4% in NFHS-4 (2015-2016) [9].

Iron deficiency is the most frequent cause of anemia, accounting for 50-60% of cases, followed by deficiency of folate and vitamin B12 [1-2]. Iron deficiency is multifactorial and can be either due to reduced iron availability from diet due to insufficient dietary iron intake or poor absorption, or due to increased losses from vomiting or blood loss further compounded by increased iron demands in pregnancy [10]. The Indian diet is essentially a cereal diet containing either rice or wheat that has non-heme iron with high phytate levels, which inhibit iron absorption [10]. There is an inadequate intake of meat, green leafy vegetables, fruits, and eggs in the Indian diet making Indian pregnant women vulnerable to iron deficiency and anemia. The high prevalence of hookworm and other worm infestations further compounds anemia [10].

The Minimum Dietary Diversity for Women (MDD-W) score is a useful tool to examine the consumption of 10 food items in the diet, showing whether women are consuming diverse foods or not [11,12]. Diverse foods are useful and cause less malnutrition and anemia, while less diverse foods (only cereals with some milk, vegetables, and pulses) are risk factors for anemia in pregnancy.

The present study was conducted to observe the consumption of 10 diverse foods in the diet and its correlation with the prevalence and severity of anemia in pregnancy in a tertiary referral hospital.

## Materials and methods

This was a cross-sectional study. A total of 430 pregnant women in their second and third trimester of pregnancy attending the antenatal clinic of Vardhman Mahavir Medical College and Safdarjang Hospital New Delhi, India, between January 2023 and September 2023 were recruited for the study. Written informed consent was taken from all participants. The study was approved by the Institutional Ethical Committee of Vardhman Mahavir Medical College and Safdarjang Hospital (approval number: IEC/VMMC/SJH/thesis/06/2022/Cc-T1 dated July 11, 2022).

Taking the prevalence of anemia in pregnancy to be 42% as per NFHS-5 [8] with a 10% non-response rate, the sample size was calculated to be 430. Patients with obstetrics complications like antepartum hemorrhage, pre-eclampsia, and co-morbidities like diabetes mellitus, hypertension, and cardiac disease were excluded from the study to avoid any confounding factors.

About five to eight antenatal patients were enrolled from each antenatal clinic using systematic random sampling. They were interviewed as per proforma containing questions about sociodemographic, obstetric, and nutritional factors including MDD-W score. The socioeconomic status was classified as per the modified Kuppuswamy classification with updated income criteria in 2022 [13].

Definition of anemia

A hemoglobin (Hb) concentration of less than 11 gm% was taken as the definition of anemia (as per WHO)[1]. The grading of anemia in pregnancy was as follows: Mild anemia: Hb 9-10.9 gm%; Moderate anemia: Hb 7-8.9 gm%; Severe anemia: Hb 4-6.9 g%; Very severe anemia: <4 gm%.

Data analysis

The data was analyzed and statistical analysis was performed using Stata Statistical Software: Release 18 (2023; StataCorp LLC, College Station, Texas, United States). Categorical data was presented as frequency and percentage values. The association between anemia and MDD-W score was tested using the chi-square test. For all statistical tests, a two-sided possibility of p-value <0.05 was considered statistically significant.

## Results

A total of 430 pregnant women in the second and third trimesters of pregnancy were observed to see the impact of the MDD-W score on the prevalence of anemia. The characteristics of the participants are shown in Table 1. The age of the study participants ranged from 18 to 43 years, with the mean being 26.2±4.5 years, while parity ranged from 0 to 6 with the median being 2.0. BMI calculated from pre-pregnancy body weight ranged from 16.5 to 35.5 kg/m^2^ with the mean being 22.4±4.3 kg/m^2^. Socioeconomic status as per the modified Kuppuswamy classification was lower in 41 (9.53%), upper lower in 42 (9.72%), lower middle in 46 (10.78%), upper middle in 197 (45.81%), and upper class in 104 (24.19%) women (Table 2).

**Table 1:** Sociodemographic characteristics of the patients (N=430)

Characteristics	Values
Age (years), mean±SD (range)	26.2±4.5 (18-43)
Parity, median	2
BMI (kg/m^2^), mean±SD (range)	22.4±4.3 (16.5-35.5)

**Table 2:** Socioeconomic status of patients (N=430)

Socioeconomic status	Frequency	Percentage
Lower	41	9.53%
Upper lower	42	9.72%
Lower middle	46	10.78%
Upper middle	197	45.81%
Upper	104	24.19%

The grading of anemia as per WHO under the study is given in Table 3. Hb >11g/dl was considered normal while Hb <11g/dl was considered as anemia. The prevalence and severity of anemia are shown in Table 4. Out of the total 430 women, 210 (48.84%) women had anemia in pregnancy, with mild anemia in 111 (25.81%), moderate anemia in 68 (15.81%), severe anemia in 30 (6.98%), and very severe anemia in one woman (0.24%). The dietary habits with respect to vegetarian and non-vegetarian diets are shown in Table 5. A total of 212 (49.3%) women were vegetarian, out of which 116 (55.24%) were anemic as compared to 218 (50.69%) non-vegetarians among whom only 94 (44.7%) were anemic (p=0.04). Hence, a vegetarian diet was a risk factor for anemia.

**Table 3:** Grading of anemia in pregnancy

S. No.	Grading	Hemoglobin (gm/dl)
1	Normal	11 and above
2	Anaemia	<11
3	Mild anaemia	9-10.9
4	Moderate anaemia	7-8.9
5	Severe anaemia	4-6.9
6	Very severe (decompensated) anaemia	<4

**Table 4:** Prevalence and severity of anemia in the study population Hb: hemoglobin

Groups according to Hb levels	Number of Patients	Percentage out of Total Patients (N=430)	Percentage out of Anemic Patients (N=210)
Normal Hb	220	51.16%	-
Total Anemia	210	48.84%	100%
Mild Anemia	111	25.81%	52.86%
Moderate Anemia	68	15.81%	32.38%
Severe Anemia	30	6.98%	14.29%
Very Severe Anemia	1	0.24%	0.47%

**Table 5:** Comparison of anemia grades between vegetarian and non-vegetarian patients

Diet	Normal (n=220), n (%)	Mild Anemia (n=111), n (%)	Moderate Anemia (n=68), n (%)	Severe Anemia (n=31), n (%)	Total Anemia (n=210), n (%)	Total (n=430), n (%)	P value	Significance
Vegetarian	96 (43.64%)	59 (53.15%)	38 (55.88%)	19 (61.29%)	116 (55.24%)	212 (49.3%)	0.04	S
Non-Vegetarian	124 (56.36%)	52 (46.85%)	30 (44.12%)	12 (38.71)	94 (44.76%)	218 (50.69%)	0.04	S

Daily dietary calories and protein and iron intake in the non-anemia and anemia groups are shown in Table 6. Daily calorie intake was 1105-3157 kcal with the mean being 1982±362.4 Kcal in the non-anemia group in contrast to 897-3025 kcal with the mean being 1637±298.3 in the anemia group (p=0.02). Daily dietary protein intake ranged from 32 to 114 gm with the mean being 68.4±6.8gm in the non-anemia group in contrast to 15.5-102 gm with a mean of 50.2±5.1 in the anemia group (p=0.015). The MDD-W food groups are shown in Table 7. They were grains, white roots and tubers, and plantains in Group 1, pulses (beans, peas, and lentils) in Group 2, nuts and seeds in Group 3, dairy products (milk and milk products) in Group 4, meat, poultry and fish in Group 5, eggs in Group 6, dark green leafy vegetables in Group 7, vitamin A rich fruits and vegetables in Group 8, other vegetables in Group 9, and other fruits in Group 10.

**Table 6:** Daily dietary calories and protein and iron intake in the anemia and non-anemia groups (N=430)

Characteristics	Non-anemia group (n=220), Mean ± SD (range)	Anemia group (n=210), Mean ± SD (range)	P-value	Significance
Calories	1982±362.4 (1105-3157)	1637±298.3 (897-3025)	0.02	S
Proteins (gm/dl)	68.4±6.8 (32-114)	50.2±5.1 (15.5-102)	0.015	S
Dietary iron (mg)	33.2±3.41 (15-60)	21.1±1.97 (8-58)	0.015	S

**Table 7:** MDD-W food groups and score according to comsumption MDD-W: Minimum Dietary Diversity for Women

S. No.	MDD-W Food Groups	Consumption ≥ 15 g/day	Consumption <15g/day
1	Grains, white roots and tubers, and plantains	1	0
2	Pulses (beans, peas and lentils)	1	0
3	Nuts and seeds	1	0
4	Dairy	1	0
5	Meat, poultry, and fish	1	0
6	Eggs	1	0
7	Dark green leafy vegetables	1	0
8	Other vitamin A-rich fruits and vegetables	1	0
9	Other vegetables	1	0
10	Other fruits	1	0

The MDD-W score in the patients in the present study with its relationship to the prevalence and severity of anemia is shown in Table 8. The overall MDD-W score ranged from 2 to 10 with the mean being 5.7±1.1. MDD-W score ranged from 4 to 10 with the mean being 6.2±1.2 in the normal (non-anemia group) while it ranged from 2 to 8 with the mean being 3.8±0.75 in the anemia group with a significant difference (p= 0.01). On further breakup, the MDD-W score ranged from 3 to 8 with the mean being 4.4±0.9 in mild anemia (normal vs mild anemia, p=0.02, significant) while it ranged from 2 to 7 with the mean being 3.5±0.7 in the moderate anemia group (normal vs moderate anemia, p=0.05, significant). In severe anaemia, the MDD-W score ranged from 2 to 6 with the mean being 2.2±0.45 (normal vs severe anaemia, p=0.001, highly significant). Hence MDD-W score was significantly lower in the anemia group than in the normal hemoglobin group. The MDD-W score was lowest in the severe anemia group.

**Table 8:** MDD-W score in the different groups (N=430) MDD-W: Minimum Dietary Diversity for Women

	Normal (n=220)	Mild (n=111)	Moderate (n=61)	Severe (n=31)	Anaemia (n=210)	Total (N=430)
Score range	4-10	3-8	2-7	2-6	2-8	2-10
Score mean±SD	6.2±1.2	4.2±0.9	3.5±0.7	2.2±0.45	3.8±0.75	5.7±1.1

## Discussion

Anaemia is the most common disease, affecting more than 1.5 billion people globally, with a much higher prevalence in developing nations like India [1-3]. The highest prevalence of anemia is seen in children aged 2-5 years, women of reproductive age group, and pregnant women [1-3]. Pregnant women are the most vulnerable group to anemia and pose a significant adverse effect on maternal and perinatal outcomes [10,14]. Stevens et al. performed a pooled analysis of population-representative data for national regional and global estimates of anemia in women for 2000-2019 and observed the prevalence of anemia in pregnancy to be 36.5% globally, 5.7% in the United States, and 75% in Africa and Asia [15]. Various Indian studies showed the prevalence of anemia in pregnancy to be 65-95% [4-8]. The prevalence of anemia in the present study was found to be 48.84% which was less than the national average of 52.2% but more than 42% of Delhi in the NFHS-5.

Iron deficiency anemia (IDA) is the commonest cause of anemia, responsible for 50-60% of cases [3]. The reason for IDA is due to reduced iron availability from the diet by insufficient iron intake or poor absorption from cereal diet with nonheme iron and excess phytates, which are inhibitors of iron absorption [10,14].

The MDD-W score was introduced by the Food and Agriculture Organization of the United Nations in 2016 [11]. It was further updated in 2021 [12]. The MDD-W was developed as a proxy indicator to reflect the micronutrient adequacy of women’s diets at national and international levels [11,12]. It’s a population-level indicator based on recall of single day and night diet from individual women. It takes into consideration 10 food items which include grains, white roots and tubers, and plantains (group 1), pulses, beans, peas, and lentils (group 2), nuts and seeds (group 3), dairy products (milk and milk products, (group 4), meat, poultry, and fish (group 5), eggs (group 6), dark green leafy vegetables (group 7), other vitamin A rich fruits and vegetables (group 8), other vegetables (group 9), and other fruits (group 10) [11,12].

Validation studies have shown that women consuming food items from at least five of the groups are likely to have higher micronutrient adequacy than women who consume food from fewer groups [16]. The women who consume food items from five or more of the 10 food groups are also highly likely to consume at least one animal source food and other pulses or nuts and seeds and food items from two or more of the fruits and vegetables food groups [16]. Women consuming red and processed meat had a significantly higher intake of micronutrients [17]. Eggs of birds (domesticated poultry and wild birds) are a good source of proteins, vitamins, especially B12, and a range of other bioavailable micronutrients [17]. Groups 7 to 10 are vegetables and fruits, which are rich sources of micronutrients and vitamins, especially folate and vitamin B12, which prevent micronutrient deficiency [18]. Group 4 food items (milk and milk products) are an important source of high-quality proteins, potassium, calcium, and vitamins, especially B12 and other micronutrients, and are especially crucial for vegetarian women which is a common scenario in India where a significant percentage of women are vegetarian (49.3% women in the present study were vegetarians).

In the present study, the prevalence of anemia was 48.84% (25.84% mild, 15.85% moderate, and 7.2% severe). The overall mean MDD-W score was 5.7±1.1, being significantly more (6.2±1.2) in the normal Hb group than in the anemia group (3.8±0.75). The mean score was 4.9±0.9 in the mild anemia group, 3.5±0.7 in the moderate anemia group, and only 2.2±0.45 in the severe anemia group.

Dietary diversity has been studied by various authors in pregnancy and was found to be inversely related to pre-pregnancy BMI [19]. Yang et al. observed low (<5) MDD-W score in pregnancy to be associated with less gestational weight gain and adverse birth outcomes [20]. Etea et al. observed mediating effects of women’s empowerment on dietary diversity during pregnancy in central west Ethiopia and advocated better education for women as an empowerment tool for household decision-making with a higher likelihood of consuming a more diverse diet with improved maternal and perinatal outcomes [21]. Zerfu et al. also observed dietary diversity during pregnancy to be associated with reduced risk of maternal anemia, preterm deliveries, and low birth weight [22].

Other studies also observed dietary diversity and associated factors amongst pregnant women [23-29]. Sinharoy et al. observed that women’s dietary diversity in rural Bangladesh could be improved through women’s empowerment with improved maternal and perinatal outcomes [30]. In a study from rural Odisha, India, Jin et al. observed 30% lower odds of mild anemia (p=0.035) with a more diverse diet [31]. In another cross-sectional study from Prayagraj, Uttar Pradesh, India, Saggu et al. observed the prevalence of anemia to be 47.4%, of which only 18.1% of women consumed a diverse diet and 81.89% had low MDD-W score [32]. Manjula et al. observed a positive impact of dietary diversity on the nutritional status of pregnant women in Bijapul, North Karnataka, India [33]. Rao et al. observed increased mean Hb with consumption of diverse diets in rural Maharashtra, India [34].

Limitations

This was a hospital-based study and hence doesn’t give the true prevalence of anemia in the community. The women were asked about dietary history in the previous 24 hours with a risk of recall bias. Another limitation was that it was a one-time study with no follow-up regarding maternal and perinatal outcomes.

## Conclusions

The MDD-W score is a useful tool to examine the consumption of 10 major food items like cereals, pulses, nuts, dairy, meat, poultry, fish, dark green leafy vegetables, vitamin A-rich fruits, and other varieties of fruits and vegetables. It helps to elucidate whether patients are consuming foods with varied diversity like meat, eggs, vegetables, and fruits to prevent IDA.

An MDD-W score of 5 or more is indicative of the diversity of food with less prevalence of anemia while an MDD-W score of <5 is an indicator of poor diversity with a higher prevalence of anemia as proven in the present study. Hence, pregnant women should be advised to consume diverse foods in their diet.
